# Unraveling the role of resistin, retinol-binding protein 4 and adiponectin produced by epicardial adipose tissue in cardiac structure and function: evidence of a paracrine effect

**DOI:** 10.1007/s42000-023-00447-5

**Published:** 2023-03-24

**Authors:** Georgios A. Christou, Christina E. Andriopoulou, Alexandra Liakopoulou, Eirini Tsape, Efstratios Apostolakis, Alexandros D. Tselepis, Maria Konstandi, Gema Frühbeck, Dimitrios N. Kiortsis

**Affiliations:** 1 Atherothrombosis Research Centre, Medical School, University of Ioannina, 45110 Ioannina, Greece; 2 Department of Pharmacology, Medical School, University of Ioannina, Ioannina, Greece; 3 Department of Cardiac Surgery, University Hospital of Ioannina, Ioannina, Greece; 4 Atherothrombosis Research Centre/Laboratory of Biochemistry, Department of Chemistry, University of Ioannina, Ioannina, Greece; 5 Department of Endocrinology and Nutrition, Clínica Universidad de Navarra, Pamplona, Spain; 6 Metabolic Research Laboratory, University of Navarra, Pamplona, Spain

**Keywords:** Adipokines, Epicardial fat, Adipose tissue, Left ventricular function, Coronary artery disease

## Abstract

**Purpose:**

Adipokines produced by adipose tissue have been found to be involved in the pathophysiology of metabolic and cardiovascular diseases. We aimed to investigate the relationships of resistin, retinol-binding protein 4 (RBP4) and adiponectin produced by epicardial adipose tissue with coronary artery disease (CAD) and cardiac structure and function.

**Methods:**

Forty-one non-diabetic males scheduled for cardiothoracic surgery were examined. Anthropometric measurements, echocardiography, coronary angiography, and blood analysis were performed preoperatively. We measured the serum levels of resistin, RBP4, and adiponectin and their mRNA expression in thoracic subcutaneous adipose tissue and two epicardial adipose tissue samples, one close to left anterior descending artery (LAD) (resistin-LAD, RBP4-LAD, adiponectin-LAD), and another close to the right coronary artery (RCA) (resistin-RCA, RBP4-RCA, adiponectin-RCA).

**Results:**

Left ventricular (LV) ejection fraction correlated negatively with adiponectin-LAD (rho =  − 0.390, *p* = 0.025). The ratio of early to late diastolic transmitral flow velocity, as an index of LV diastolic function, correlated negatively with resistin-LAD (rho =  − 0.529, *p* = 0.024) and RBP4-LAD (rho =  − 0.458, *p* = 0.049). There was no difference in epicardial adipose tissue mRNA expression of resistin, RBP4, and adiponectin between individuals with CAD and those without CAD. When we compared the individuals with CAD in the LAD with those without CAD in the LAD, there was no difference in resistin-LAD, RBP4-LAD, and adiponectin-LAD. There was no difference in resistin-RCA, RBP4-RCA, and adiponectin-RCA between the individuals with CAD in the RCA and those without CAD in the RCA.

**Conclusion:**

Elevation of epicardial adipose tissue mRNA expression of adiponectin was associated with LV systolic dysfunction, while that of both resistin and RBP4 was linked to LV diastolic dysfunction.

**Supplementary Information:**

The online version contains supplementary material available at 10.1007/s42000-023-00447-5.

## Introduction

A growing body of evidence suggests that the adipokines produced by adipose tissue may be involved in the pathophysiology of metabolic and cardiovascular diseases [1–5]. Serum levels of specific adipokines, including leptin, adiponectin, resistin, and retinol-binding protein 4 (RBP4), have been associated with the presence of coronary artery disease (CAD), suggesting a potential role of these adipokines in the pathogenesis of CAD [1, 3–5]. Moreover, increased serum levels of leptin, resistin, and RBP4 and decreased levels of adiponectin have previously been reported to be associated with left ventricular (LV) dysfunction and the development of heart failure, highlighting the importance of these adipokines in the pathophysiology of heart failure [5–12].

Deeper investigation into the role of the adipokines produced by epicardial adipose tissue in cardiac disease is warranted, given that these cytokines are highly likely to exert an impact on the neighboring myocardium through a paracrine effect. Studies to date on the relationship between epicardial adipose tissue mRNA expression of adipokines and cardiovascular disease have yielded conflicting results, which could be attributed to the fact that these studies have focused almost exclusively on CAD, essentially neglecting the potential role of the adipokines produced by epicardial adipose tissue in the cardiac structure and function [13–16]. Convincing evidence points to an association of epicardial adipose tissue mRNA expression of leptin with LV diastolic function; meanwhile, the roles of epicardial adipose tissue mRNA expression of resistin, RBP4, and adiponectin in cardiac structure and function remain to be elucidated [7]. The above studies, moreover, did not take into account the possibility that regional epicardial adipose tissue mRNA expression of adipokines could be associated with the presence of significant stenosis in the corresponding coronary artery or with the structure and function of the corresponding myocardium. We postulated that epicardial adipose tissue mRNA expression of resistin, RBP4, and adiponectin from epicardial areas corresponding to the LV myocardium may correlate with echocardiographic indices of LV systolic and diastolic function. Additionally, we hypothesized that epicardial adipose tissue mRNA expression of resistin, RBP4, and adiponectin from epicardial areas close to major coronary arteries could correlate with the severity of atherosclerosis of the corresponding coronary artery. Thus, the aim of the present study was to investigate the relationships of resistin, RBP4, and adiponectin produced by different regions of epicardial adipose tissue with cardiac structure and function and with CAD.

## Materials and methods

### Subjects

In the present study, 41 non-diabetic males scheduled for cardiothoracic surgery were consecutively recruited from the cardiothoracic clinic of the University Hospital of Ioannina, Ioannina, Greece. Exclusion criteria were as follows: age less than 18 years, severe kidney disease, liver disease, gastrointestinal disease, malignancy, any endocrine, or metabolic disorder (e.g., Cushing’s syndrome, untreated hypothyroidism, or hyperthyroidism) other than obesity, more than 5% change in body weight (BW) during the 3 months prior to enrollment, any state of systemic inflammation and treatment with antidiabetic or antiobesity drugs and nonsteroidal anti-inflammatory agents, or glucocorticoids, within the preceding 3 weeks. Diagnosis of diabetes mellitus as an exclusion criterion was based on previous taking of antidiabetic medications or fasting blood glycemia (i.e., ≥ 126 mg/dL on two separate tests).

All subjects were evaluated by an experienced clinician. A detailed medical history was obtained, and a comprehensive physical examination was carried out. The subjects’ height and BW were measured. Body surface area was calculated using the Du Bois formula. Body fat percentage (BF%) was estimated using the CUN-BAE (Clínica Universidad de Navarra-Body Adiposity Estimator) formula [17]. Body fat mass (BFM) was calculated as BW × BF%/100 and lean body mass as BW – BFM. An individual was diagnosed with CAD if there was evidence of at least one coronary artery stenosis ≥ 50% on preoperative coronary angiography. Venous sampling was performed 1 day prior to surgery. Collection of venous blood samples was carried out in the morning, following an overnight fast of at least 12 h. After centrifugation, the serum samples were stored at − 80 °C until analysis.

Three biopsy samples were collected from each patient before extracorporeal circulation was initiated. The three biopsy samples included one subcutaneous adipose tissue sample from the thoracic region, one epicardial adipose tissue sample close to the left anterior descending artery (LAD) and another epicardial adipose tissue sample close to the right coronary artery (RCA). The samples were transported in normal saline solution. After centrifugation, the fat pads for mRNA determination were stored at − 80 °C until analysis.

Written informed consent was obtained from all the study participants. This study was performed in accordance with the Declaration of Helsinki and was approved by the Research Ethics Committee of the University Hospital of Ioannina (741/2017). The trial was registered in ClinicalsTrials.gov (NCT03322332).

### Quantitative real-time polymerase chain reaction

The TRIzol reagent Invitrogen was used for the isolation of total RNA from subcutaneous and epicardial (close to the LAD or the RCA) white adipose tissue samples from patients who underwent cardiac surgery. A spectrophotometric method was applied for the determination of total RNA concentration in each sample. A SuperScript II reverse trancriptase kit (Invitrogen) and total RNA (1 μg) from each sample were utilized for cDNA production, which was used in quantitative and reverse transcription polymerase chain reaction (PCR) assays. The sequences of the forward and reverse gene-specific primers that were used in this study are displayed in Supplementary Table 1. SYBR Green PCR Master Mix (Applied Biosystems, Foster City) was used for real-time reactions, which were performed using the Thermal Cycler Real-Time Detection System C1000 (BioRad, Italy). The relative mRNA expression levels of each gene were normalized using β-actin mRNA levels (QuantiTect primer assay, QIAGEN) and the values were quantified using the comparative threshold cycle method. The following abbreviations were used for the mRNA expression of adipokines: resistin-sc, RBP4-sc, and adiponectin-sc: mRNA expression of resistin, RBP4, and adiponectin from subcutaneous adipose tissue; resistin-LAD, RBP4-LAD, and adiponectin-LAD: mRNA expression of resistin, RBP4, and adiponectin from epicardial adipose tissue samples close to the LAD; and resistin-RCA, RBP4-RCA, and adiponectin-RCA: mRNA expression of resistin, RBP4, and adiponectin from epicardial adipose tissue samples close to the RCA. Quantitative real-time polymerase chain reaction (qPCR) was performed in duplicate for each adipose tissue sample.

### ELISA measurements

Measurement of serum resistin was performed with an ELISA kit (R&D Systems, Inc., Minneapolis, USA) [18]. The intra-assay coefficient of variation (CV) was < 6% and the inter-assay CV was < 10%.

Serum RBP4 was analyzed using an ELISA kit (ALPCO Diagnostics, Salem, MA, USA) [19, 20]. The intra-assay CV was 5% and the inter-assay CV was < 10%.

Serum adiponectin was determined using an ELISA kit (ALPCO DIAGNOSTICS, Salem, NH, USA) [21]. The intra-assay CV was 5% and the inter-assay CV was 5%.

### Blood test measurements

Biochemical parameters were determined from fresh blood samples by standard laboratory methods. Low-density lipoprotein cholesterol (LDL-C) was calculated using the Friedewald equation LDL-C = TC − HDL-C—TG/5 (TC: total cholesterol, HDL-C: high density lipoprotein cholesterol, TG: serum triglycerides) in individuals with TG ≤ 400 mg/dL. Non-HDL-cholesterol was calculated as TC—HDL-C. The estimated glomerular filtration rate was calculated based on the Chronic Kidney Disease Epidemiology Collaboration (CKD-EPI) formula.

### Echocardiography

All echocardiographic images were acquired using a commercially available ultrasound system (Vivid I; GE Medical; Horten, Norway) with a 1.5- to 4-MHz phased-array transducer. All images were obtained by the same experienced cardiologist-ultrasonographer with the participant in the left lateral decubitus position. A comprehensive assessment of the structure and function of the left heart was undertaken by the same cardiologist in accordance with the guidelines of the American Society of Echocardiography and the European Association of Cardiovascular Imaging [22]. LV end-diastolic volume (LVEDV) and end-systolic volume were estimated using the Simpson biplane method, allowing the calculation of LV ejection fraction (LVEF). Both early (MVE) and late (MVA) diastolic transmitral flow velocities were measured via the use of pulsed wave Doppler echocardiography with the sample volume placed in the tips of the mitral valve. The ratio of early to late diastolic transmitral flow velocity (MVE/A) was calculated. Mitral annular velocities were measured at the septal and lateral aspects of the mitral valve annulus using tissue Doppler. The ratio of MVE to the average of septal and lateral early diastolic mitral annular velocity [MVEa(s-l)] was used as an estimate of LV filling pressures. LV stiffness was estimated by the ratio MVE/Ea(s-l)/LVEDV [23].

### Statistical analysis

All statistical analyses were performed using the software IBM SPSS Statistics 23.0. The Shapiro–Wilk test was used to verify the normality of the distributions of the parameters of interest. Parameters with normal distribution were expressed as mean ± standard deviation and with non-normal distribution as median (minimum–maximum). Comparisons between two independent groups were performed with the independent *t*-test for normally distributed variables and the Mann–Whitney *U* test for non-normal variables. Comparisons between more than two independent groups were performed with the Kruskal–Wallis H test. Before correlation analysis, parameters with non-normal distribution were subjected to logarithmic transformation if the parameters were characterized by positive skew or power transformation (second power) in the case of negative skew. Logarithmic transformation of parameters with non-normal distribution was performed only to better identify the outliers. Analysis for exclusion of outliers using Cook’s distance (values with Cook’s distance > 4/n were considered outliers, where n was the number of observations) preceded correlation analysis. The univariate associations between the parameters of interest were assessed with Spearman’s correlation analysis. We performed backward stepwise regression analysis to predict LVEF and MVE/A using as independent variables age, body mass index (BMI), the categorical variable for the existence of CAD or not, logadipokine-LAD, and log(circulating adipokine). A two-tailed *p* value < 0.05 was considered statistically significant.

## Results

### Characteristics of the study participants

The median age was 66 (38–83) years and the mean BMI was 28.4 ± 3.5 kg/m^2^ (17 overweight and 16 obese individuals) (Table 1). The reasons for cardiac surgery were as follows: (*n* = 13) angina, (*n* = 14) recent myocardial infarction, (*n* = 9) valvular heart disease, (*n* = 2) newly diagnosed heart failure, and (*n* = 3) dilatation of the thoracic aorta. Thirty individuals were diagnosed with CAD, including one patient with one-vessel disease, 14 patients with two-vessel disease and 15 patients with three-vessel disease. CAD in the LAD was found in 29 patients, while CAD in the RCA was detected in 21 patients. Twenty-eight individuals were diagnosed with arterial hypertension and one with atrial fibrillation. The biochemical parameters are presented in Supplementary Table 2.

**Table 1:** Characteristics of the study participants

	All (*n* = 41)	No CAD (*n* = 11)	CAD (*n* = 30)
Age (years)	66 (38–83)	66 (39–81)	66 (38–83)
BW (kg)	82.6 ± 12.3	83.0 ± 13.0	82.4 ± 12.2
BMI (kg/m^2^)	28.4 ± 3.5	28.2 ± 3.8	28.5 ± 3.5
BF% (%)	30.7 ± 4.1	30.5 ± 4.5	30.8 ± 4.0
Lean/overweight/obese	6/17/16 (not available data for 2 patients)	3/4/4	3/13/12
CAD	30	0	30
1VD/2VD/3VD	1/14/15	0/0/0	1/14/15
Hypertension	28	10	18
Atrial fibrillation	1	1	0
COPD	4	1	3
Never/former/current smokers	5/14/19 (not available data for 3 patients)	2/3/5	3/11/14
Regular alcohol drinkers	19	6	13
Moderate/excessive alcohol use	7/12	3/3	4/9
Statins	27	3	24
b-blocker	26	4	22
RAAS inhibitors	25	10	15

*BF%* body fat percentage, *BMI* body mass index, *BW* body weight, *CAD* coronary artery disease, *COPD* chronic obstructive pulmonary disease, *RAAS* renin–angiotensin–aldosterone system, *1VD* one-vessel disease, *2VD* two-vessel disease, *3VD* three-vessel disease

Data are expressed as mean ± SD for normally distributed variables or median (minimum–maximum) for non-normally distributed variables

Moderate alcohol use was defined as consumption of up to two drinks per day

### Comparisons of adipokines between groups

Table 2 shows the comparisons of adipose tissue mRNA expression and serum levels of adipokines between individuals with CAD and no CAD. There was no difference in subcutaneous and epicardial adipose tissue mRNA expression of resistin, RBP4, and adiponectin between individuals with CAD and those without CAD. Serum adiponectin levels were lower in patients with CAD compared to patients without CAD (*p* < 0.001). The statistical significance of the comparisons of adipose tissue mRNA expression and serum adipokine levels between individuals with CAD and no CAD was not influenced after adjustment for statin treatment.

**Table 2:** Adipose tissue mRNA expression and serum levels of adipokines in individuals with coronary artery disease (CAD) and no CAD

	All (*n* = 41)	No CAD (*n* = 11)	CAD (*n* = 30)	*p* value (CAD vs No CAD)
Resistin-sc	2.52 (0.01–287.27)	4.74 (0.10–287.27)	1.93 (0.01–96.72)	0.201
Resistin-LAD	1.90 (0.01–186.03)	0.74 (0.01–186.03)	3.82 (0.03–121.86)	0.251
Resistin-RCA	0.35 (0.01–136.62)	0.78 (0.06–136.62)	0.35 (0.01–56.87)	0.126
Circulating resistin (ng/mL)	5.99 (3.80–13.37)	7.65 (4.55–13.37)	5.93 (3.80–11.52)	0.147
RBP4-sc	6.32 (0.15–129.95)	5.42 (0.60–129.95)	6.35 (0.15–69.64)	0.837
RBP4-LAD	4.21 (0.01–764.97)	3.56 (0.01–39.30)	4.64 (0.12–764.97)	0.195
RBP4-RCA	0.10 (0.01–2.34)	0.09 (0.01–2.34)	0.10 (0.01–0.67)	0.724
Circulating RBP4 (mg/L)	13.31 ± 1.55	13.93 ± 1.19	12.99 ± 1.64	0.102
Adiponectin-sc	0.21 (0.01–4.00)	0.71 (0.01–2.58)	0.15 (0.01–4.00)	0.052
Adiponectin-LAD	7.40 (0.01–687.77)	3.24 (0.01–687.77)	15.16 (0.20–338.67)	0.356
Adiponectin-RCA	0.58 (0.03–22.51)	0.58 (0.09–22.51)	0.70 (0.03–20.57)	0.848
Circulating adiponectin (mg/L)	3.06 (1.77–12.89)	6.55 (2.34–12.89)	2.50 (1.77–5.82)	** < 0.001**

Data are expressed as mean ± standard deviation for normally distributed variables or median (minimum–maximum) for non-normally distributed variables. Comparisons between individuals with CAD and the ones without CAD were performed with independent *t*-test for normally distributed variables and Mann–Whitney *U* test for non-normal variables. *P* values in bold indicate statistically significant differences

*CAD* coronary artery disease, *LAD* left anterior descending artery, *RBP4* retinol-binding protein 4, *RCA* right coronary artery, *sc* subcutaneous

When we compared the individuals with significant CAD in the LAD with those without significant CAD in the LAD, there was no difference in resistin-LAD (*p* = 0.229), RBP4-LAD (*p* = 0.218) and adiponectin-LAD (*p* = 0.426). There was no difference in resistin-RCA (*p* = 0.230), RBP4-RCA (*p* = 0.774), and adiponectin-RCA (*p* = 0.322) between the individuals with significant CAD in the RCA and those without significant CAD in the RCA.

### Correlation analysis of adipokines

#### Associations of adipokines with echocardiographic parameters

The associations of adipose tissue mRNA expression of adipokines with echocardiographic parameters are detailed in Table 3. Left ventricular end-diastolic internal diameter correlated negatively with RBP4-LAD. LVEF was negatively associated with adiponectin-LAD. MVE/A correlated negatively with resistin-LAD and RBP4-LAD. Aortic valve peak velocity (AVVmax) was positively associated with resistin-RCA, RBP4-RCA, and adiponectin-RCA. RBP4-LAD correlated positively with MVE/Ea(s-l)/LVEDV (rho = 0.762, *p* = 0.028), an estimate of LV stiffness.

**Table 3:** Echocardiographic parameters in individuals subjected to cardiac surgery and their associations with epicardial adipose tissue mRNA expression of adipokines

		Resistin-LAD		Resistin-RCA		RBP4-LAD		RBP4-RCA		Adiponectin-LAD		Adiponectin-RCA	
		rho	*p* value	rho	*p* value	rho	*p* value	Rho	*p* value	rho	*p* value	rho	*p* value
LVIVSd (cm)	1.0(0.8–1.6)	− 0.058	0.782	0.252	0.259	0.213	0.318	− 0.028	0.900	− 0.199	0.341	0.180	0.399
LVPWTd (cm)	1.0(0.8–1.5)	− 0.001	0.998	0.062	0.785	− 0.116	0.607	0.051	0.821	− 0.050	0.825	0.348	0.113
LVIDd (cm)	5.3 ± 0.8	− 0.131	0.552	− 0.327	0.127	− **0.665**	**0.001**	0.211	0.358	0.013	0.952	0.040	0.852
LVEDVi (mL/m^2^)	70.7 ± 21.6	− 0.153	0.485	− 0.330	0.116	− **0.611**	**0.002**	0.012	0.958	− 0.016	0.943	0.015	0.942
LVEF (%)	55(33–65)	0.119	0.503	− 0.112	0.527	− 0.173	0.352	− 0.006	0.975	− **0.390**	**0.025**	− 0.096	0.585
LAd (cm)	4.0 ± 0.5	0.211	0.385	0.082	0.739	0.390	0.099	0.144	0.555	0.070	0.781	0.002	0.994
MVE/A	0.80 (0.57–2.25)	− **0.529**	**0.024**	− 0.230	0.329	− **0.458**	**0.049**	0.155	0.514	− 0.074	0.764	− 0.082	0.737
MVE/Ea(s-l)	9.8 ± 1.9	0.297	0.405	0.418	0.229	0.300	0.433	− 0.527	0.117	0.050	0.898	− 0.018	0.960
Aroot (cm)	3.3 ± 0.6	0.156	0.488	0.049	0.824	0.049	0.833	0.182	0.405	− 0.253	0.269	**0.466**	**0.029**
AVVmax (m/s)	1.7 (0.7–5.5)	0.191	0.447	**0.578**	**0.015**	− 0.178	0.495	**0.494**	**0.044**	− 0.212	0.430	**0.796**	** < 0.001**

Data are expressed as mean ± SD for normally distributed variables or median (minimum–maximum) for non-normally distributed variables. The univariate associations between the parameters of interest were assessed with Spearman’s correlation analysis. Values in bold indicate statistically significant associations

*Aroot* aortic root diameter, *AVVmax* aortic valve peak velocity, *LAD* left anterior descending artery, *LAd* left atrial diameter, *LVEDVi* left ventricular end-diastolic volume indexed to body surface area, *LVEF* left ventricular ejection fraction, *LVIDd* left ventricular end-diastolic internal diameter, *LVIVSd* left ventricular interventricular septum thickness at end-diastole, *LVPWTd* left ventricular posterior wall thickness at end-diastole, *MVE/A* ratio of early to late diastolic transmitral flow velocity, *MVE/Ea(s-l)* ratio of the early diastolic transmitral flow velocity to the average of septal and lateral early diastolic mitral annular velocity, *RBP4* retinol-binding protein 4, RCA: right coronary artery

### Multiple linear regression analysis

#### LV systolic function

We performed backward stepwise regression analysis to predict LVEF using as independent variables age, BMI, the categorical variable for the existence of CAD or not, logadiponectin-LAD, and log(circulating adiponectin). The final model included only one independent variable, logadiponectin-LAD (standardized β =  − 0.382, *p* = 0.050).

#### LV diastolic function

We performed backward stepwise regression analysis to predict MVE/A using as independent variables age, BMI, the categorical variable for the existence of CAD or not, logresistin-LAD, and log(circulating resistin). The final model (adjusted R^2^ = 0.336, *p* = 0.012) included BMI (standardized β =  − 0.345, *p* = 0.092) and logresistin-LAD (standardized β =  − 0.455, *p* = 0.031) as independent variables. Thus, the only independent determinant of MVE/A was logresistin-LAD.

We performed backward stepwise regression analysis to predict MVE/A using as independent variables age, BMI, the categorical variable for the existence of CAD or not, logRBP4-LAD, and RBP4-serum. The final model (adjusted R^2^ = 0.389, *p* = 0.006) included BMI (standardized β =  − 0.366, *p* = 0.061) and logRBP4-LAD (standardized β =  − 0.500, *p* = 0.014) as independent variables. Therefore, the only independent determinant of MVE/A was logRBP4-LAD.

## Discussion

The key findings of the present study include the following: (1) increased adiponectin mRNA expression from LV epicardial adipose tissue was associated with reduced LV systolic function, whereas elevation of both resistin and RBP4 mRNA expression from LV epicardial adipose tissue was linked with decreased LV diastolic function; (2) the regional epicardial adipose tissue mRNA expression of resistin, RBP4, and adiponectin did not differ between individuals with CAD of the corresponding coronary artery and those without CAD.

### Associations of adipokines with LV systolic function

An inverse relationship was found in the present study between LVEF and adiponectin mRNA expression from LV epicardial adipose tissue, implying an upregulation of the production of adiponectin from LV epicardial adipose tissue in patients with LV systolic dysfunction. Indeed, cardiac release of adiponectin was previously reported to correlate positively with brain natriuretic peptide levels and negatively with LVEF in patients with reduced LVEF [24]. Furthermore, an inverse association between plasma adiponectin levels and LVEF and an elevation of plasma adiponectin levels in individuals with systolic heart failure have previously been shown [10–12]. Considering the fact that natriuretic peptides have been reported to enhance adiponectin production in patients with heart failure and adiponectin has been demonstrated to exert beneficial effects on myocardial systolic function through an antioxidant mechanism and AMP-activated protein kinase (AMPK)-dependent production of vascular endothelial growth factor, elevation of adiponectin production from epicardial adipose tissue may represent a counterregulatory response to myocardial insufficiency [24–27]. Notably, the current study found that LVEF was associated only with adiponectin-LAD, but not with adiponectin-RCA, suggesting that the upregulation of the production of adiponectin from the epicardial adipose tissue may occur only locally corresponding to the LV myocardial segments with systolic dysfunction.

### Associations of adipokines with LV diastolic function

We demonstrated that increased levels of both resistin-LAD and RBP4-LAD were associated with a delayed relaxation pattern of LV filling, as indicated by decreased MVE/A. Importantly, LV diastolic dysfunction was associated only with epicardial adipose tissue mRNA expression of adipokines from the region of the LAD, which corresponds to the LV myocardium, highlighting the possible existence of a causal relationship. In this respect, resistin and RBP4 secreted by the epicardial adipose tissue of the LV myocardium may promote the development of increased LV chamber stiffness resulting in diastolic dysfunction of delayed relaxation pattern. Consistently, serum levels of both resistin and RBP4 have previously been reported to be associated with LV diastolic dysfunction and development of heart failure, highlighting the importance of these adipokines in the pathogenesis of heart failure [6–9]. The mechanism underlying resistin-induced myocardial dysfunction was recently elucidated by Zhao et al. using experiments in mice [28]. Specifically, deletion of resistin in vivo was demonstrated to attenuate the overload-induced LV myocardial dysfunction, whereas overexpression of resistin was shown to deteriorate cardiac function and induce heart failure in mice through mechanisms involving the miR148b-3p/Gadd45a axis and DNA damage response [28]. With regard to the mechanism of RBP4-induced myocardial dysfunction, Zhang et al. demonstrated that knockdown of RBP4 in the heart diminished acute myocardial dysfunction-related cardiac dysfunction via downregulation of cardiomyocyte pyroptosis [29]. The present study demonstrates for the first time, to our knowledge, the relationship of LV diastolic dysfunction with epicardial adipose tissue mRNA expression of resistin and RBP4, indicating the possible existence of a paracrine effect of these adipokines on the neighboring myocardium. This paracrine effect may exist independently from any systemic effect of serum resistin and RBP4, as suggested by the results of the backward stepwise regression analysis of this study.

### Associations of adipokines with hemodynamics of aortic valve

AVVmax, which increases in relation to the severity of calcific aortic stenosis, was found in this study to correlate positively with mRNA expression of resistin, RBP4, and adiponectin from epicardial adipose tissue near the RCA. Taking into account that the RCA passes through the right atrioventricular groove, which is in close proximity to the fibrous skeleton of the heart, the epicardial adipose tissue specimens near the RCA were collected from a region adjacent to the fibrous skeleton of the heart, the calcification of which is expected to be increased in individuals with aortic valve calcification [30]. Therefore, the extensive calcification of the epicardial adipose tissue near the RCA may upregulate the mRNA expression of resistin, RBP4, and adiponectin in cases of calcific aortic stenosis. This postulated mechanism could be further corroborated by the previously reported induction of adiponectin expression in adipocytes by the calcification-promoting hormone osteocalcin and the association of vascular expression of adiponectin with vascular calcification [31, 32].

### Associations of adipokines with CAD

The comparison of epicardial adipose tissue mRNA expression and serum levels of resistin, RBP4, and adiponectin between individuals with CAD and without CAD revealed a significant difference only for serum adiponectin levels. Indeed, circulating adiponectin has previously been shown to be downregulated in patients with CAD and involved in anti-atherosclerotic pathways [1, 33]. The inconsistency in the reported associations of epicardial adipose tissue mRNA expression of resistin, RBP4, and adiponectin with CAD in the literature may challenge the notion that these adipokines produced by epicardial adipose tissue could be causally linked with coronary atherosclerosis through a paracrine effect [13–16, 34, 35]. Considering that the patients with advanced CAD who underwent coronary artery bypass surgery in these studies were expected to be more commonly characterized by LV diastolic and systolic dysfunction compared to individuals without CAD, the reported associations of epicardial adipose tissue mRNA expression of resistin, RBP4, and adiponectin with CAD in some studies could be attributed at least in part to the association of these adipokines with LV dysfunction [36, 37]. Consistently, among patients with CAD, epicardial adipose tissue mRNA expression of resistin was found to be upregulated only in those with a history of myocardial infarction or those with acute coronary syndrome, implying the possible existence of the confounding effect of LV dysfunction [13, 34]. Furthermore, epicardial adipose tissue mRNA expression of adiponectin was demonstrated to be downregulated only in patients with multivessel CAD and not in individuals with one-vessel disease [35]. The present study demonstrated for the first time that regional (i.e., close to the LAD or the RCA) epicardial adipose tissue mRNA expression of adipokines is not associated with the presence of significant stenosis of the coronary artery supplying the relevant myocardial vascular region, thus further reinforcing the concept of a possible neutral paracrine role of these adipokines produced by epicardial adipose tissue in coronary atherosclerosis.

### Study strengths and limitations

Strengths of the present study include, firstly, the fact that we applied a novel approach to determine whether regional (i.e., close to the LAD or the RCA) epicardial adipose tissue mRNA expression of adipokines may be associated with the presence of significant stenosis in the corresponding coronary artery or with the structure and function of the corresponding myocardium. Secondly, as opposed to the overwhelming majority of previous relevant studies, the population of the current study included only non-diabetic participants, eliminating the confounding effect of diabetic status on the study of adipokines [4].

The results of our study should be interpreted in light of some limitations. Firstly, a proportion of participants were evaluated in the period following a recent myocardial infarction, which may have altered mRNA expression of adipokines from epicardial adipose tissue samples in close proximity to a healing myocardial infarction. Secondly, the ranges of distributions of adipokine mRNA expressions were relatively wide. Nevertheless, the relevant parameters were evaluated after log-transformation, resulting in less non-normal distribution with decreased variability. A similarly wide range of epicardial adipose tissue mRNA expression of adipokines has previously been reported [38]. Indeed, skewed distribution with the presence of outliers is increasingly being recognized as a not uncommon characteristic of human gene expression, attributed to various potential mechanisms, including gene interaction and incomplete penetrance [39]. Thirdly, taking into account the cross-sectional design of the current study, the observed relationships between epicardial adipose tissue mRNA expression of adipokines and the studied parameters cannot prove the presence of causal associations. Moreover, a proportion of the study population was taking statins that may have influenced the adipokine mRNA expressions. Fifthly, it should be acknowledged that the non-parametric tests that were used in the present study are generally characterized by lower power than the respective parametric tests. Furthermore, the number of patients with CAD was greater than the number of individuals without CAD resulting in decreased power of the study to detect significant differences. These two facts together may represent a limitation of this study as concerns the associated increase in type II error. In this respect, further studies with greater numbers of participants are needed to confirm the absence of statistically significant differences of the studied parameters.

## Conclusion

**Fig. 1:**
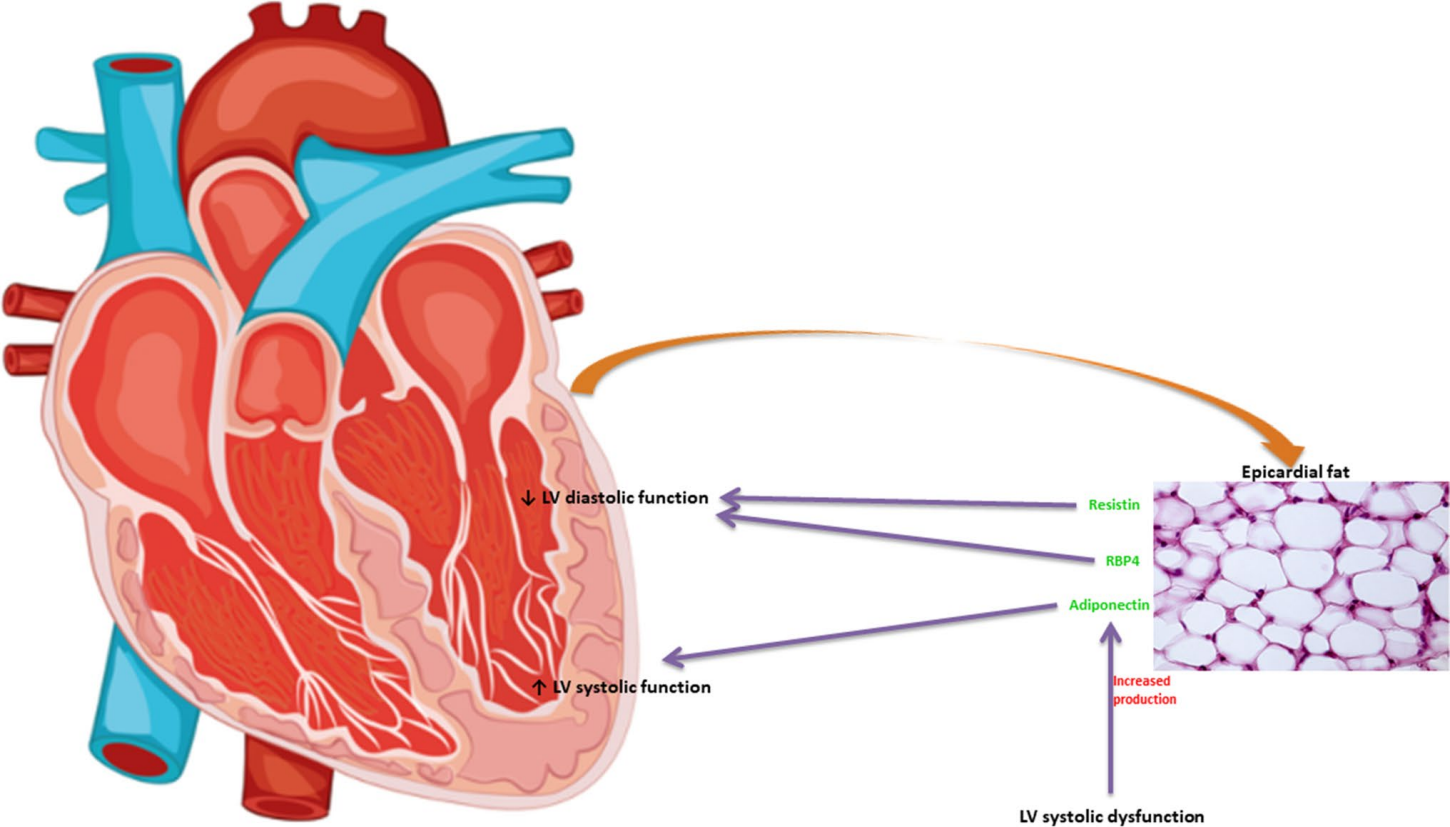
Associations of resistin, retinol-binding protein 4 (RBP4), and adiponectin produced by epicardial adipose tissue with left ventricular (LV) myocardial function

In conclusion, increased epicardial adipose tissue mRNA expression of adiponectin was associated with LV systolic dysfunction, while the elevation of epicardial adipose tissue mRNA expression of both resistin and RBP4 was associated with LV diastolic dysfunction (Fig. 1). Further studies are required to elucidate whether these associations represent causal relationships and if the adipokines produced by epicardial adipose tissue can be promising targets of future treatments for heart failure.

## Supplementary Information

Below is the link to the electronic supplementary material.

Supplementary Tables 1 and 2 (DOCX 25 kb)

Supplementary Figure 1. Box plot of logmRNA expressions of resistin, retinol-binding protein 4 (RBP4) and adiponectin in subcutaneous adipose tissue (sc), epicardial adipose tissue close to left anterior descending artery (LAD) and epicardial adipose tissue close to right coronary artery (RCA). Data values that are at least 1.5 times the interquartile range larger than the third quartile or at least 1.5 times the interquartile range smaller than the first quartile are denoted outliers (tiny circles). (PNG 293 kb)

High resolution image (TIF 134 kb)

## Data Availability

Data available on request.

## References

[1] Pischon T, Girman CJ, Hotamisligil GS, Rifai N, Hu FB, Rimm EB (2004). Plasma adiponectin levels and risk of myocardial infarction in men. JAMA.

[2] Christou GA, Kiortsis DN (2013). Adiponectin and lipoprotein metabolism. Obes Rev.

[3] Muse ED, Feldman DI, Blaha MJ, Dardari ZA, Blumenthal RS, Budoff MJ, Nasir K, Criqui MH, Cushman M, McClelland RL, Allison MA (2015). The association of resistin with cardiovascular disease in the Multi-Ethnic Study of Atherosclerosis. Atherosclerosis.

[4] Christou GA, Tselepis AD, Kiortsis DN (2012). The metabolic role of retinol binding protein 4: an update. Horm Metab Res.

[5] Wannamethee SG, Shaper AG, Whincup PH, Lennon L, Sattar N (2011). Obesity and risk of incident heart failure in older men with and without pre-existing coronary heart disease: does leptin have a role?. J Am Coll Cardiol.

[6] Porcar-Almela M, Codoñer-Franch P, Tuzón M, Navarro-Solera M, Carrasco-Luna J, Ferrando J (2015). Left ventricular diastolic function and cardiometabolic factors in obese normotensive children. Nutr Metab Cardiovasc Dis.

[7] Toczylowski K, Hirnle T, Harasiuk D, Zabielski P, Lewczuk A, Dmitruk I, Ksiazek M, Sulik A, Gorski J, Chabowski A, Baranowski M (2019). Plasma concentration and expression of adipokines in epicardial and subcutaneous adipose tissue are associated with impaired left ventricular filling pattern. J Transl Med.

[8] Butler J, Kalogeropoulos A, Georgiopoulou V, de Rekeneire N, Rodondi N, Smith AL, Hoffmann U, Kanaya A, Newman AB, Kritchevsky SB, Vasan RS, Wilson PW, Harris TB (2009). Serum resistin concentrations and risk of new onset heart failure in older persons: the health, aging, and body composition (Health ABC) study. Arterioscler Thromb Vasc Biol.

[9] Chavarria N, Kato TS, Khan R, Chokshi A, Collado E, Akashi H, Takayama H, Naka Y, Farr M, Mancini D, Schulze PC (2012). Increased levels of retinol binding protein 4 in patients with advanced heart failure correct after hemodynamic improvement through ventricular assist device placement. Circ J.

[10] Cavusoglu E, Chopra V, Battala V, Ruwende C, Yanamadala S, Eng C, Pinsky DJ, Marmur JD (2008). Baseline plasma adiponectin levels as a predictor of left ventricular systolic dysfunction in patients referred for coronary angiography. Am J Cardiol.

[11] Tang WH, Shrestha K, Tong W, Wang Z, Troughton RW, Borowski AG, Klein AL, Hazen SL (2013). Nitric oxide bioavailability and adiponectin production in chronic systolic heart failure: relation to severity of cardiac dysfunction. Transl Res.

[12] Tengiz İ, Türk UÖ, Alioğlu E, Kırılmaz B, Tamer GS, Tüzün N, Ercan E (2013). The relationship between adiponectin, NT-pro-BNP and left ventricular ejection fraction in non-cachectic patients with systolic heart failure: an observational study. Anadolu Kardiyol Derg.

[13] Langheim S, Dreas L, Veschini L, Maisano F, Foglieni C, Ferrarello S, Sinagra G, Zingone B, Alfieri O, Ferrero E, Maseri A, Ruotolo G (2010). Increased expression and secretion of resistin in epicardial adipose tissue of patients with acute coronary syndrome. Am J Physiol Heart Circ Physiol.

[14] Salgado-Somoza A, Teijeira-Fernández E, Rubio J, Couso E, González-Juanatey JR, Eiras S (2012). Coronary artery disease is associated with higher epicardial retinol-binding protein 4 (RBP4) and lower glucose transporter (GLUT) 4 levels in epicardial and subcutaneous adipose tissue. Clin Endocrinol (Oxf).

[15] Jaffer I, Riederer M, Shah P, Peters P, Quehenberger F, Wood A, Scharnagl H, März W, Kostner KM, Kostner GM (2012). Expression of fat mobilizing genes in human epicardial adipose tissue. Atherosclerosis.

[16] Iacobellis G, di Gioia CR, Cotesta D, Petramala L, Travaglini C, De Santis V, Vitale D, Tritapepe L, Letizia C (2009). Epicardial adipose tissue adiponectin expression is related to intracoronary adiponectin levels. Horm Metab Res.

[17] Gómez-Ambrosi J, Silva C, Catalán V, Rodríguez A, Galofré JC, Escalada J, Valentí V, Rotellar F, Romero S, Ramírez B, Salvador J, Frühbeck G (2012). Clinical usefulness of a new equation for estimating body fat. Diabetes Care.

[18] Christou KA, Christou GA, Karamoutsios A, Vartholomatos G, Gartzonika K, Tsatsoulis A, Tigas S (2020). The regulation of serum resistin levels in metabolically healthy and unhealthy obese individuals. Hormones (Athens).

[19] Christou GA, Tellis CC, Elisaf MS, Tselepis AD, Kiortsis DN (2012). The changes in plasma retinol-binding protein 4 levels are associated with those of the apolipoprotein B-containing lipoproteins during dietary and drug treatment. Angiology.

[20] Christou GA, Tellis CC, Elisaf MS, Tselepis AD, Kiortsis DN (2016). The relationship between retinol-binding protein 4 and apolipoprotein B-containing lipoproteins is attenuated in patients with very high serum triglycerides: a pilot study. Hormones (Athens).

[21] Christou GA, Tellis KC, Elisaf MC, Tselepis AD, Kiortsis DN (2012). High density lipoprotein is positively correlated with the changes in circulating total adiponectin and high molecular weight adiponectin during dietary and fenofibrate treatment. Hormones (Athens).

[22] Lang RM, Badano LP, Mor-Avi V, Afilalo J, Armstrong A, Ernande L, Flachskampf FA, Foster E, Goldstein SA, Kuznetsova T, Lancellotti P, Muraru D, Picard MH, Rietzschel ER, Rudski L, Spencer KT, Tsang W, Voigt JU (2015). Recommendations for cardiac chamber quantification by echocardiography in adults: an update from the American Society of Echocardiography and the European Association of Cardiovascular Imaging. J Am Soc Echocardiogr.

[23] Kasner M, Sinning D, Burkhoff D, Tschöpe C (2015). Diastolic pressure-volume quotient (DPVQ) as a novel echocardiographic index for estimation of LV stiffness in HFpEF. Clin Res Cardiol.

[24] Takano H, Obata JE, Kodama Y, Kitta Y, Nakamura T, Mende A, Kawabata K, Saito Y, Fujioka D, Kobayashi T, Yano T, Sano K, Kugiyama K (2009). Adiponectin is released from the heart in patients with heart failure. Int J Cardiol.

[25] Tsukamoto O, Fujita M, Kato M, Yamazaki S, Asano Y, Ogai A, Okazaki H, Asai M, Nagamachi Y, Maeda N, Shintani Y, Minamino T, Asakura M, Kishimoto I, Funahashi T, Tomoike H, Kitakaze M (2009). Natriuretic peptides enhance the production of adiponectin in human adipocytes and in patients with chronic heart failure. J Am Coll Cardiol.

[26] Essick EE, Wilson RM, Pimentel DR, Shimano M, Baid S, Ouchi N, Sam F (2013). Adiponectin modulates oxidative stress-induced autophagy in cardiomyocytes. PLoS ONE.

[27] Shimano M, Ouchi N, Shibata R, Ohashi K, Pimentel DR, Murohara T, Walsh K (2010). Adiponectin deficiency exacerbates cardiac dysfunction following pressure overload through disruption of an AMPK-dependent angiogenic response. J Mol Cell Cardiol.

[28] Zhao B, Bouchareb R, Lebeche D (2022). Resistin deletion protects against heart failure injury by targeting DNA damage response. Cardiovasc Res.

[29] Zhang KZ, Shen XY, Wang M, Wang L, Sun HX, Li XZ, Huang JJ, Li XQ, Wu C, Zhao C, Liu JL, Lu X, Gao W (2021). Retinol-binding protein 4 promotes cardiac injury after myocardial infarction via inducing cardiomyocyte pyroptosis through an interaction with NLRP3. J Am Heart Assoc.

[30] Barasch E, Gottdiener JS, Larsen EK, Chaves PH, Newman AB, Manolio TA (2006). Clinical significance of calcification of the fibrous skeleton of the heart and aortosclerosis in community dwelling elderly. The Cardiovascular Health Study (CHS). Am Heart J.

[31] Lee NK, Sowa H, Hinoi E, Ferron M, Ahn JD, Confavreux C, Dacquin R, Mee PJ, McKee MD, Jung DY, Zhang Z, Kim JK, Mauvais-Jarvis F, Ducy P, Karsenty G (2007). Endocrine regulation of energy metabolism by the skeleton. Cell.

[32] Aubert CE, Liabeuf S, Amouyal C, Kemel S, Lajat-Kiss F, Lacorte JM, Halbron M, Carlier A, Salem JE, Funck-Brentano C, PerisicMatic L, Witasp A, Stenvinkel P, Phan F, Massy ZA, Hartemann A, Bourron O (2019). Serum concentration and vascular expression of adiponectin are differentially associated with the diabetic calcifying peripheral arteriopathy. Diabetol Metab Syndr.

[33] Fujishima Y, Maeda N, Matsuda K, Masuda S, Mori T, Fukuda S, Sekimoto R, Yamaoka M, Obata Y, Kita S, Nishizawa H, Funahashi T, Ranscht B, Shimomura I (2017). Adiponectin association with T-cadherin protects against neointima proliferation and atherosclerosis. FASEB J.

[34] Rachwalik M, Zyśko D, Diakowska D, Kustrzycki W (2014). Increased content of resistin in epicardial adipose tissue of patients with advanced coronary atherosclerosis and history of myocardial infarction. Thorac Cardiovasc Surg.

[35] Eiras S, Teijeira-Fernández E, Shamagian LG, Fernandez AL, Vazquez-Boquete A, Gonzalez-Juanatey JR (2008). Extension of coronary artery disease is associated with increased IL-6 and decreased adiponectin gene expression in epicardial adipose tissue. Cytokine.

[36] Lin FY, Zemedkun M, Dunning A, Gomez M, Labounty TM, Asim M, Horn E, Aurigemma G, Maurer MS, Roman M, Devereux R, Min JK (2013). Extent and severity of coronary artery disease by coronary CT angiography is associated with elevated left ventricular diastolic pressures and worsening diastolic function. J Cardiovasc Comput Tomogr.

[37] Ahmadi N, Mao SS, Hajsadeghi F, Hacioglu Y, Flores F, Gao Y, Ebrahimi R, Budoff M (2011). Relation of subclinical left and right ventricular dysfunctions measured by computed tomography angiography with the severity of coronary artery disease. Coron Artery Dis.

[38] Shibasaki I, Nishikimi T, Mochizuki Y, Yamada Y, Yoshitatsu M, Inoue Y, Kuwata T, Ogawa H, Tsuchiya G, Ishimitsu T, Fukuda H (2010). Greater expression of inflammatory cytokines, adrenomedullin, and natriuretic peptide receptor-C in epicardial adipose tissue in coronary artery disease. Regul Pept.

[39] Mar JC (2019). The rise of the distributions: why non-normality is important for understanding the transcriptome and beyond. Biophys Rev.

