# Supplementary material for: Unraveling the role of resistin, retinol-binding protein 4 and adiponectin produced by epicardial adipose tissue in cardiac structure and function: evidence of a paracrine effect
Source: Hormones (Athens). 2023 Mar 24;22(2):321–30. doi: 10.1007/s42000-023-00447-5 (PMC10209261; doi:10.1007/s42000-023-00447-5)
Supplement: Supplementary file 1 — (DOCX 25 kb) [file 42000_2023_447_MOESM1_ESM.docx]

**Supplementary Table 1.** The sequences of the forward and reverse gene-specific primers that were used in quantitative real-time polymerase chain reaction.

| **Primer** | **Sequence** |
| --- | --- |
| Human resistin F | 5'-TGCAGGATGAAAGCTCTCTGT-3' |
| Human resistin R | 5'-TGGCAGTGACATGTGGTCTC-3' |
| Human RBP4 F | 5'-CTTGCGCGCGGTTCCC-3’ |
| Human RBP4 R | 5'-TTGACTCGGAAGCTGCTCAC-3' |
| Human Adiponectin F | 5’-TGGTGAGAAGGGTGAGAA-3’ |
| Human Adiponectin R | 5’-AGATCTTGGTAAAGCGAATG-3’ |

**Abbreviations:** F: forward, RBP4: retinol-binding protein 4, R: reverse.

**Supplementary Table 2.** Biochemical parameters in individuals subjected to cardiac surgery.

|  | **All (n=41)** | **No CAD (n=11)** | **CAD (n=30)** |
| --- | --- | --- | --- |
| **Glucose (mg/dL)** | 101±14 | 100±11 | 103±15 |
| **TC (mg/dL)** | 165(107-295) | 188(167-252) | 147(107-295) |
| **TG (mg/dL)** | 156(61-401) | 152(81-335) | 159(61-401) |
| **HDL-C (mg/dL)** | 33(20-83) | 41(24-83) | 33(20-51) |
| **LDL-C (mg/dL)** | 92(55-228) | 109(85-173) | 81(55-228) |
| **nonHDL-C (mg/dL)** | 126(81-262) | 157(101-207) | 113(81-262) |
| **Creatinine (mg/dL)** | 1.07±0.23 | 1.03±0.14 | 1.08±0.25 |
| **eGFR (mL/min/1.73m^2^)** | 76±18 | 78±14 | 76±19 |
| **TSH (μIU/mL)** | 1.37(0.19-3.83) | 1.16(0.19-3.12) | 1.38(0.38-3.83) |
| **FT_4_ (ng/dL)** | 0.88±0.13 | 0.87±0.10 | 0.89±0.15 |
| **CRP (mg/L)** | 4(1-83) | 4(2-16) | 4(1-83) |

Data are expressed as mean ± SD for normally distributed variables or median (minimum-maximum) for non-normally distributed variables.

**Abbreviations:** CAD: coronary artery disease, CRP: C-reactive protein, eGFR: estimated glomerular filtration rate, FT4: free thyroxine, HDL-C: high density lipoprotein cholesterol, LDL-C: low density lipoprotein cholesterol, nonHDL-C: non high density lipoprotein cholesterol, TC: total cholesterol, TG: serum triglycerides, TSH: thyroid-stimulating hormone.
