# Supplementary figures and images for: Unraveling the role of resistin, retinol-binding protein 4 and adiponectin produced by epicardial adipose tissue in cardiac structure and function: evidence of a paracrine effect
Source: Hormones (Athens). 2023 Mar 24;22(2):321–30. doi: 10.1007/s42000-023-00447-5 (PMC10209261; doi:10.1007/s42000-023-00447-5)

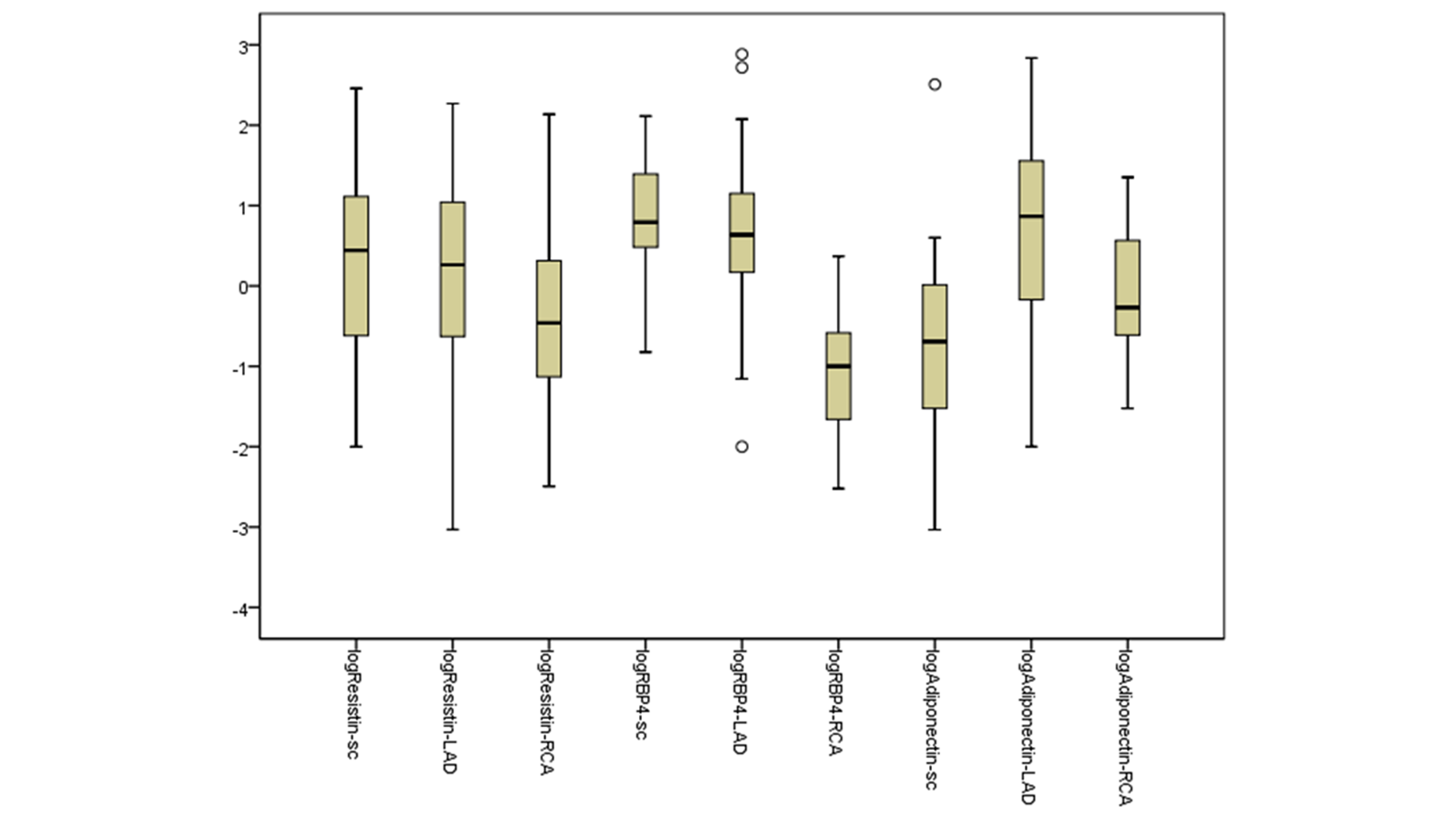

Supplement: Supplementary file 2 — Box plot of logmRNA expressions of resistin, retinol-binding protein 4 (RBP4) and adiponectin in subcutaneous adipose tissue (sc), epicardial adipose tissue close to left anterior descending artery (LAD) and epicardial adipose tissue close to right coronary artery (RCA). Data values that are at least 1.5 times the interquartile range larger than the third quartile or at least 1.5 times the interquartile range smaller than the first quartile are denoted outliers (tiny circles). (PNG 293 kb) [file 42000_2023_447_Fig2_ESM.png]

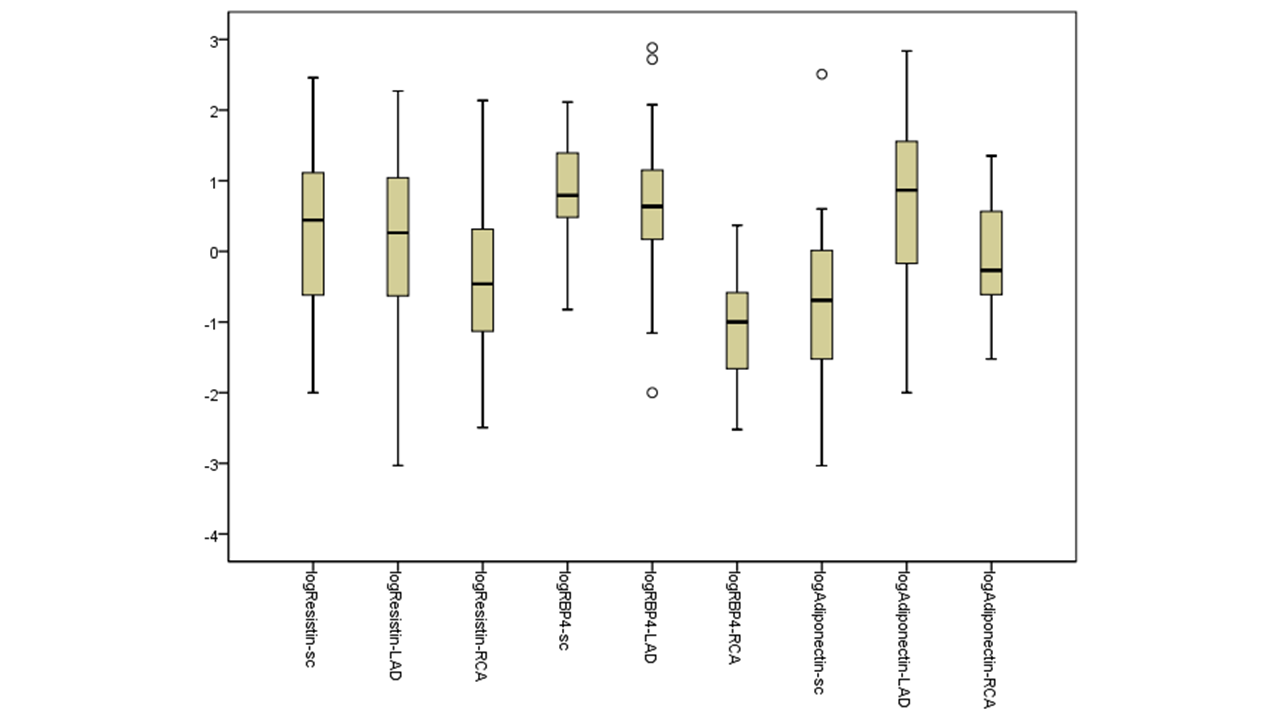

Supplement: Supplementary file 3 — High resolution image (TIF 134 kb) [file 42000_2023_447_MOESM2_ESM.tif]
